# Claudin-1 required for HCV virus entry has high potential for phosphorylation and *O*-glycosylation

**DOI:** 10.1186/1743-422X-8-229

**Published:** 2011-05-15

**Authors:** Waqar Ahmad, Khadija Shabbiri, Bushra Ijaz, Sultan Asad, Muhammad T Sarwar, Sana Gull, Humera Kausar, Kiran Fouzia, Imran Shahid, Sajida Hassan

**Affiliations:** 1 Applied and Functional Genomics Lab, Centre of Excellence in Molecular Biology, University of the Punjab, Lahore-53700, Pakistan; 2 Department of Chemistry, GC University Lahore, Pakistan

## Abstract

HCV is a leading cause of hepatocellular carcinoma and cirrhosis all over the world. Claudins belong to family of tight junction's proteins that are responsible for establishing barriers for controlling the flow of molecules around cells. For therapeutic strategies, regulation of viral entry into the host cells holds a lot of promise. During HCV infection claudin-1 is highly expressed in liver and believed to be associated with HCV virus entry after HCV binding with or without co-receptor CD81. The claudin-1 assembly with tight junctions is regulated by post translational modifications. During claudins assembly and disassembly with tight junctions, phosphorylation is required at C-terminal tail. In cellular proteins, interplay between phosphorylation and O-*β*-GlcNAc modification is believed to be functional switch, but it is very difficult to monitor these functional and vibrant changes *in vivo*. Netphos 2.0 and Disphos 1.3 programs were used for potential phosphorylation; NetPhosK 1.0 and KinasePhos for kinase prediction; and YinOYang 1.2 and OGPET to predict possible *O*-glycosylation sites. We also identified Yin Yang sites that may have potential for O-*β*-GlcNAc and phosphorylation interplay at same Ser/Thr residues. We for the first time proposed that alternate phosphorylation and O-*β*-GlcNAc modification on Ser 192, Ser 205, Ser 206; and Thr 191 may provide an on/off switch to regulate assembly of claudin-1 at tight junctions. In addition these phosphorylation sites may be targeted by novel chemotherapeutic agents to prevent phosphorylation lead by HCV viral entry complex.

## Introduction

HCV, the deadly virus has infected almost 3% of the world population. Most of the infected patients develop chronic infection leading to end stage hepatocellular carcinoma. A better understanding of mechanism of infection and the potential host co-factors facilitating its replication is an urgent need of the hour for the release of disease burden and vaccine development [1-3].

In multicellular organisms, movement of ions, proteins and water is controlled by barriers known as tight junctions (TJs) formed by epithelial and endothelial cell monolayers [4]. While tight junctions require the coordinated activity of several different proteins, the specificity of tight junction's permeability is regulated by transmembrane proteins known as claudins [5]. Entry of HCV in to the hepatocytes is a complex process and involves interaction of HCV glycoproteins E1 and E2 with host receptor CD81 and scavenger receptor class B member I (SR-BI). It is reported that these two receptors are not sufficient for its entry and later another receptor claudin-1 was discovered which play an important role in viral entry lately after viral binding to the CD81 [6,7]. Claudins are transmembrane proteins which play important role in tight junction formation and act as barrier in cellular permeability. Tight junctions are the combination of transmembrane and peripheral proteins tied with cytoskeleton. Several classes of claudin interact with other proteins to form tight junction and regulate permeability of TJs. It is also observed that the expression of claudin proteins found to be differentially regulated in several cancers. Claudin-1 expression was observed up-regulated in liver, stomach, thyroid, pancreas and cervix tumor formations [8-12]. Claudin-1 removal in mouse epidermis results in dramatic trans-epidermal water loss, inferring its indispensable role in creating and maintaining the epidermal barrier [13].

In biological systems, protein localization, activity, their interaction with other proteins and overall turnover is determined by post translational modifications (PTMs) [14]. Several PTMs like phosphorylation, glycosylation, acetylation and methylation are some well known examples. Phosphorylation in claudin protein family is well observed and believed to be modulating TJs permeability on both charged and uncharged ligands and molecules [9,15]. Several enzymes like protein kinase A (PKA), protein kinase C (PKC), protein phosphatase 2A (PP2A), MAPK etc are involved during claudin phosphorylation [16-18]. Phosphorylation has dual effect on TJs functionality i.e. phosphorylation on some claudins increased paracellular permeability or enhanced barrier function [19]. It is reported that claudin-1 phosphorylation enhances its barrier functions while dephosphorylation leads to detergent solubility and enhanced paracellular permeability [20].

*O-*glycosylation is also very important PTM of nuclear and cytoplasmic proteins. During *O-*glycosylation one molecule of N-acetylglucosamine (*O*-GlcNAc) is introduced on Ser or Thr residue by enzyme OGT (*O-*GlcNAc transferase). Addition of O-*β*-GlcNAc can inhibit phosphorylation on Ser or Thr residue. Interplay between O-*β*-GlcNAc modification and phosphorylation on the same amino acid residues has been observed in several nuclear and cytoplasmic proteins [21]. These PTMs are dynamic and result in temporary conformational changes and regulate many functions of the proteins. The interchange of these two modifications on the same or neighboring residue may modulate the specific function of the proteins either by enhancing or inhibiting the functional capacity. Residues where O-*β*-GlcNAc and phosphorylation compete for each other are known as Yin Yang sites [22]. These Yin Yang sites can be predicted and analyzed using various computer-assisted neural network-based programs, which can help us to determine proteins regulatory functions by accessing their modification potentials. The present work describe potential phosphorylation, *O-*glycosylation and their possible interplay sites which may influence claudin-1 interaction with TJs and their possible effects on HCV entry and future therapeutics.

## Materials and methods

The FASTA sequence of human claudin-1 was retrieved from the SWISS-PROT sequence database [23] with entry name CLD1_human. The primary accession number for this sequence was O95832. Homology search was made using the BLAST at NCBI database with default parameters [24]. The search was made for all organisms' sequences. A total of 250 hits were retrieved for claudin-1 with highest bits score and zero expected values. Out of 70 retrieved sequences, seven were selected representing major mammalian or vertebrate groups. The accession numbers for eight selected sequences (Table 1) were O95832 (Human), Q6L708 (Bovine), D6RU0 (Sheep), C3VMK8 (Pig), O88551 (Mouse), P56745 (Rat), Q5ZMG2 (Chick) and Q5FW44 (Xentr). ClustalW [25] was used for multiple alignments of all the sequences of claudin-1 to get the conservation status.

**Table 1:** Different claudin-1 proteins used for multiple alignment

Species name	Universal name	**Accession no**.	Identity	Score	E-Value
*Homo sapiens*	Human	O95832	100%	-	-

*Bos tauurus*	Bovine	Q6L708	92.0%	1,053	1.0 × 10-113

*Ovis aries*	Sheep	D6R6U0	91.0%	1,049	1.0 × 10-112

*Sus scrofa*	Pig	C3VMK8	92.0%	1,018	1.0 × 10-109

*Mus musculus*	Mouse	O88551	90.0%	1,025	1.0 × 10-109

*Rattus novergicus*	Rat	P56745	91.0%	1,030	1.0 × 10-110

*Gallus gallus*	Chick	Q5ZMG2	74.0%	875	3.0 × 10-92

*Xenopus tropicalis*	Xentr	Q5FW44	67.0%	800	1.0 × 10-83

The claudin-1 sequence used in this study was "MANAGLQLLGFILAFLGWIGAIVSTALPQWRIYSYAGDNIVTAQAMYEGLWMSCVSQSTGQIQCKVFDSLLNLSSTLQATRALMVVGILLGVIAIFVATVGMKCMKCLEDDEVQKMRMAVIGGAIFLLAGLAILVATAWYGNRIVQEFYDPMTPVNARYEFGQALFTGWAAASLCLLGGALLCCSCPRKTTSYPTPRPYPKPAPSSGKDYV".

### Post-translational modifications prediction methods

We used more than one bioinformatics tools to access the post-translational modification on claudin-1 to get best results.

#### Prediction of phosphorylation residues and related kinases

Phosphorylation potential for human claudin-1 was predicted by using NetPhos 2.0 [26] and Disphos 1.3 server [27]. These are neural network-based programs that predict the potential phosphorylation sites for each Thr, Ser and Tyr residues. The minimum threshold value used to predict phosphorylation is 0.5 for NetPhos 2.0.

Kinase specific phosphorylation sites in human claudin-1 were predicted by NetPhosK 1.0 [28] and KinasePhos 2.0 server [29]. These servers predict the kinase specific acceptor substrates including Ser, Thr and Tyr.

For the evaluation of experimentally verified phosphorylation sites on human claudin-1, Phospho.ELM database was used [30]. This database contains a collection of experimentally confirmed Ser, Thr and Tyr residues in eukaryotic proteins.

#### Prediction of o-glycosylated residues and Yin Yang sites

*O-β*-GlcNAc modification potential sites were predicted by YinOYang 1.2 [31-34] and OGPET [35]. YinOYang 1.2 program can predict the potential phosphorylation sites as well and hence predict the Yin Yang sites with highly uneven threshold that is adjusted in accordance with amino acid surface accessibility. The potential Yin Yang sites can also be predicted using this method.

#### Protein structure analysis

As there is no template model of claudin-1 available in protein data bank [36], we designed an ab-initio model by using software I-TASSER [37]. Data in sequence form was uploaded to the server. Model with high C-score was selected as ab-initio model. To view and analyze 3D structure Jmol [38] and PYmol [39] programs were used. To assess, whether the predicted Ser and Thr residues have surface accessibility for post-translational modifications, NetSurfP was used [40].

#### Neural networks-based prediction methods

All the methods used for predicting post translational modifications like ANNs (artificial neural networks) or SVM (support vector machine) etc have been extensively used in biological sequence study and predicting the possible potentials for PTMs [41]. The methods developed using machine learning approach includes memorizing the neural networks with the sequence environment windows of phosphorylated/glycosylated and non-phosphorylated/non-glycosylated sites. The input data of phosphorylated/glycosylated and non-phosphorylated/non-glycosylated sites is presented to the neural networks in the form of binary codes of 21 digits. A threshold value in the form of bits is set for positive hit and zero for negative hits. The learning process and performance is checked with the data reserved for cross validation using statistical equations. During learning, the error is computed and weights given to each neuron are set to get the maximum correct predictions. It helps in reducing the error and hence decreasing the false positive and false negative prediction sites.

## Results

### Alignment of sequences for the determination of conserved status of Ser/Thr residues within claudin-1

**Figure 1:**
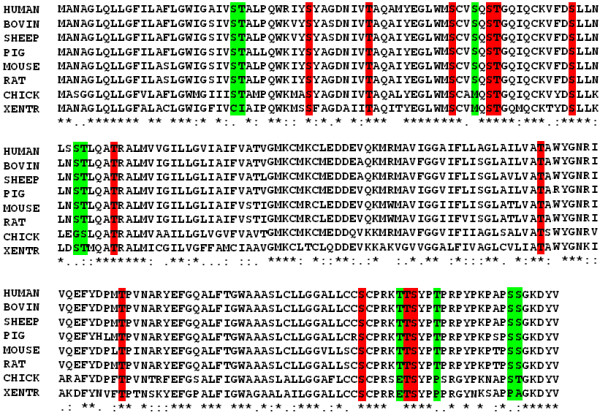
**Multiple alignments of seven vertebrates sequences (Human, Bovine, Sheep, Pig, Mouse, Rat, Chick and Xentr)**. These sequences were ordered as in aligned results from ClustalW. The consensus sequence is marked by an asterisk, conserved substitution by a double dot, and semiconserved substitution by a single dot.

Human claudin-1 was aligned with other species. Conserved and semi-conserved substituted Ser and Thr residues within each subtype were determined (Figure 1). It is clear from figure that Ser 34, 53, 58, 69, 185 and 192; and Thr 42, 59, 80, 153 and 191 are highly conserved in vertebrates. Meanwhile, Ser 24, 56, 75, 173, 205 and 206; and Thr 25, 76, 99, 167, 190 and 195 are conserved in mammals.

### Acquiring experimentally verified S/T/Y residues

Data for experimentally confirmed S/T/Y residues was obtained from Phospho.ELM and UniprotKB http://www.uniprot.org. Human claudin-1 has three phosphorylation sites Tyr 203, Ser 205 and Ser 206 by similarity with *Mus musculus*.

### Prediction of Phosphorylation Sites

**Figure 2:**
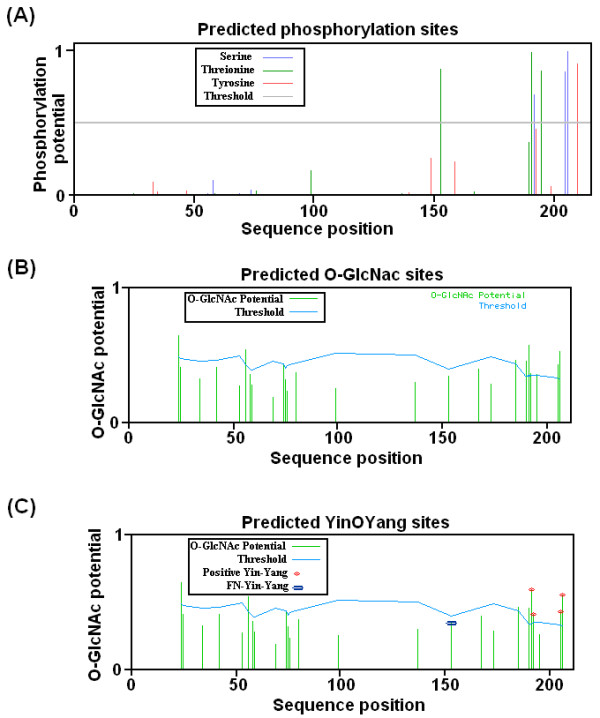
**Graphic representation of the potential Ser, Thr, and Tyr residues for phosphorylation and O-glycosylation modification for human claudin-1**. **A) **Predicted potential sites for phosphate modification on Ser and Thr residues. The light gray horizontal line indicates the threshold for modification potential. The blue, green and red vertical lines showed the potential phosphorylated Ser, Thr and Tyr residues, respectively. **B) **Predicted potential sites for O-glycosylation modification of Ser and Thr. O-*β*-GlcNAc modification potential of Ser/Thr residues is shown by green vertical line, while the light blue wavy line indicates the threshold for modification potential. **C) **The Yin Yang sites that were positively predicted are shown with red asterisk at the top, while the NP-Yin Yang site are shown with purple asterisk on the top of vertical lines. The green vertical lines show the O-*β*-GlcNAc potential of Ser/Thr residue and the light blue horizontal wavy line indicates the threshold for modification potential.

For the prediction of possible Ser and Thr residues for potential phosphorylation, NetPhos 2.0 server was used. A total of 7 sites showed high potential for phosphorylation. Amongst these 3 were Ser, 3 Thr and 1 was Tyr. All these 7 predicted sites were highly conserved in mammals (Figure 2A). Ser 58, Thr 99 and Thr 190 showed probable potential for phosphorylation. On the other hand DisPhos 1.3 predicts Thr 191, 195 and Ser 205, 206 for high and Ser 192 for probable phosphorylation potential.

### Prediction of Kinases involved in Phosphorylation

A number of kinases may be implicated for phosphorylation of Ser and Thr residues. Almost each kinase predicted is involved in phosphorylation of two or more residues. The predicted kinases involved in phosphorylation of claudin-1 by NetPhos K, KinasePhos are shown in Table 2.

**Table 2:** Predicted phosphorylation and O-glycosylation sites on Claudin-1 protein

Substrate	Position	Phosphorylation prediction		Kinase prediction		*O*-glycosylation prediction		Surface accessibility
		
		Netphos	Disphos	NetphosK	Kinasephos	YinOYang	OGPET	NetSurfP
Ser	24	N	N	-	-	N	HP	B

Thr	25	N	N	CDC2	-	N	LP	B

Ser	34	N	N	PKA	-	N	LP	B

Thr	42	N	N	-	MDD	N	VHP	B

Ser	53	N	N	-	-	N	LP	B

Ser	56	N	N	DNAPK	PKG	N	VHP	B

Ser	58	P	N	PKC	ATM, IKK	N	HP	E

Thr	59	N	N	-	PKG	N	VHP	E

Ser	69	N	N	PKA	-	N	LP	B

Ser	74	N	N	-	CKI	N	LP	E

Ser	75	N	N	-	CKI	N	HP	E

Thr	76	N	N	-	CDK	N	LP	B

Thr	80	N	N	-	-	N	LP	B

Thr	99	P	N	PKC	-	N	LP	B

Thr	137	N	N	-	-	N	LP	B

Thr	153	Y	N	MAPK, GSK3	CDC2, MAPK	N	VHP	B

Thr	167	N	N	PKC, CDC2	-	N	VHP	B

Ser	173	N	N	PKA	-	N	LP	B

Ser	185	N	N	CDC2	-	N	LP	B

Thr	190	P	N	PKA	PKA	N	HP	E

Thr	191	Y	Y	PKG	PKC, PKA	Y	LP	E

Ser	192	Y	P	-	PKA, IKK, PKB	Y	LP	E

Thr	195	Y	Y	MAPK, CDC5	PKC, MAPK, CDK	N	VHP	E

Ser	205	Y	Y	PKG	CDC2	Y	VHP	E

Ser	206	Y	Y	PKC	-	Y	VHP	E

Y = yes (threshold > 0.5), P = probable (threshold > 0.1~0.5), N = No (threshold < 0.1), B = Buried surface, E = Exposed surface, VHP = very high potential (threshold ≥ 1.0), HP = high potential (threshold >0.8 < 1.0), LP = low potential (threshold <0.8)

### Prediction of O-linked glycosylation sites

Prediction results for *O*-linked glycosylation sites showed that claudin-1 has potential for *O-β-GlcNAc *modification (Figure 2B). YinOYang 1.2 predicted 4 potential sites for O-*β*-GlcNAc modification including Ser 192, 205 and 206; and Thr 191. All these sites were highly conserved in vertebrates. Ser 206 was replaced by Thr in chick. OGPET predicts Ser 56, 205, 206 and Thr 42, 59, 153, 167, 195 for high O-glycosylation potential.

### Identification of False-Negative Sites

The Ser and Thr residues which were not predicted to be *O-β-GlcNAc *modified but showed very high potential for phosphorylation and were close to threshold value are known as false-negative sites (FN-sites). A list of Yin Yang and FN-Yin Yang residues is given in Table 3. Two residues Thr 153 and Thr 195 was predicted as FN-residue. Thr 153 was highly conserved in vertebrates while Thr 195 was conserved in mammals (Figure 1).

**Table 3:** Proposed Ser/Thr residues for the interplay of phosphorylation and *O*-β-GlcNAc modification in human claudin-1

SUBSTRATE		Proposed Yin Yang sites	Proposed Fn-Yin Yang sites	Yin Yang sites by similarity
**Cluadin-1**	SER	192, 205, 206	-	-
	
	THR	191	195	-

### Potential Yin Yang sites for claudin-1

For the interplay of phosphorylation and *O-β-GlcNAc *modification, five possible Yin Yang sites were proposed (Figure 2C). These Yin Yang sites are proposed on the basis of conservation status of Ser/Thr residues in claudin-1. The Ser/Thr residues are also proposed for the possible interplay of phosphorylation and *O-β-GlcNAc *modification on the basis of their similarity with other species. These Ser/Thr residues which are predicted "by similarity" are not yet experimentally known in human but these are known in other species of vertebrates.

## Discussion

Among vertebrates claudin-1 has highly conserved globular domain while, less conserved N- and C-terminals. Claudin-1 also showed highly conserved status among mammals (Figure 1). The claudin tails especially C-terminal is believed to be post-translationally modified [9,16,42]. The C and N- terminals of claudin associate with a number of proteins like multi-PDZ protein MUPP1, Pals1 and Zonula occludens proteins 1, 2 and 3 [43]. Evans *et al. *(2007) found that claudin-1 was necessary for HCV entry after its binding with CD81 receptors. Recent findings showed that claudin-1 can enable cell to cell transfer of HCV [7] and the C-terminal of claudin is related to protein stability, altering protein turnover and therefore the paracellular permeability [44].

It is interesting to note that the post translational modifications regulate the TJ proteins functions. However little data is available. Phosphorylation of claudin is reported to be linked with permeability modulation of TJs [9,44]. Claudin-4 is phosphorylated on Ser-194, Thr-189, claudin-3 at Thr-192, claudin-16 at Ser-217, claudin-5 at Thr-205 and Thr-207 [45-54]. In most claudins, phosphorylation at C-terminal disrupts the functions of TJs in many cancers. It is already reported that in HCV, claudin-1 expression was high as compared to other subtypes [41-43]. In mouse claudin-1, it was observed that phosphorylation on Ser and Thr residues involved in promotion of tight-junctions functions. Mouse claudin-1 is found to be phosphorylated at Ser-205, 206; and Thr-203 [46]. Thr-203 is not present in human claudin-1 and replaced with alanine residue (Figure 1). It was also observed that claudin-1 has been phosphorylated on various Ser and Thr residues in Caco-2 cell line by PKC-θ [47]. Moreover, Ser-205 and 206 are highly conserved residues in mammals and thought to be phosphorylated in other species. To predict phosphorylation sites on human claudin-1 protein, we used two tools; NetPhos and DisPhos. The predicted phosphorylation residues are given in Table 2. It is obvious from Figure 2 that Ser192, 205 and 206; while Thr153, 191 and 195 showed high potential, while Thr-190 showed probable potential for phosphorylation. Most of the high potential sites were in C-terminal. These residues were conserved in vertebrates except Thr-195. We can speculate that these residues may be possible potential phosphorylated sites in human claudin-1.

Phosphorylation of claudins by various kinases and their impact on TJs regulation is well documented. In our study we predicted many kinases that may be involved in claudin-1 phosphorylation on Ser and Thr residues irrespective of their potential to be phosphorylated. We observed that kinases such as MAPK, CDC2, PKA, PKC, PKB, and CDK were involved in human claudin-1 phosphorylation. PKC activity was observed for claudin-1, while the other claudins are phosphorylated by PKA, PKC, MPAK and EphA2. It is also reported that suppression of kinase activity disrupts TJ formation [9,19,44-55]. These reports indicate that claudin phosphorylation on C-terminal is involved in TJs formation and their performance. It was also interesting to note that claudin phosphorylation is associated with proper barrier function, while dephosphorylation negatively regulates the TJs [48,49].

**Figure 3:**
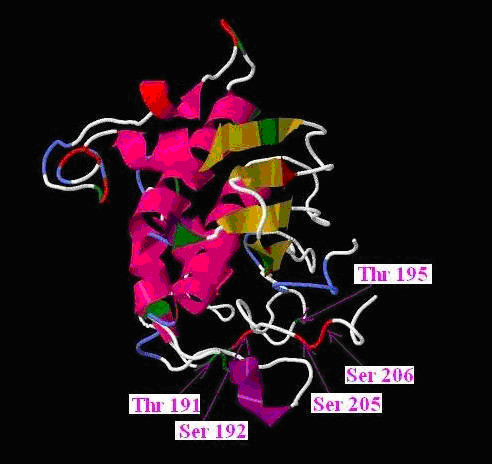
**A homology model of human claudin-1 utilizing automated protein modeling option was retrieved through I-TASSER server**. Through this option five models were received from the server utilizing five different templates namely: model 1-5. Among the five, one that covered all amino acids with alpha helix structure and beta pleated sheet, and high C-value was selected. This model showed that predicted Yin Yang sites have high surface accessibility for the phosphorylation and O-glycosylation interplay. The Ser and Thr residues are denoted by red and green colors.

*O-β-GlcNAc *modification can occur on these Ser and Thr residues where kinases are involved in phosphorylation as it is well known that kinases and OGT can compete for same site modification [32-34,56]. It is well documented that phosphorylation and O-*β*-GlcNAc modification is also a regulatory adaptation, and changes during glycosylation are transient for few hours. This shows a possibility for interplay between phosphorylation and OGT on these residues. It functions by blocking Ser/Thr residues phosphorylation and can lead to changes in protein-protein interactions, singling and protein complex arrangements. This competitive interplay is known as Yin Yang hypothesis [57-61]. Many cytoplasmic proteins undergo O-glycosylation [62]. Our prediction results showed that human claudin-1 has high potential for *O-*linked glycosylation (Figure 3, Table 2). YinOYang 1.2 server detected four, while OGPET eight Ser and Thr residues with high potential for O-glycosylation. It is clear from results that most of the residues belong to C-terminal of claudin-1. YinOYang 1.2 predicts high potential for glycosylation and their chances to become possible Yin Yang sites on Ser 192, 205 and 206; and Thr 191 (Figure 2B). Although Thr 153 and 195 has high potential for phosphorylation, these residues were not predicted to act as possible Yin Yang interplay. However, OGPET 1.0 predicts high potential for O-glycosylation on Ser 56 and, Thr 42, 59, 153, 167 and 195 based on their sequence motifs. These residues are conserved in mammals and may act as possible FN-Yin Yang sites based on their phosphorylation potential [32-34].

To predict possible Yin Yang sites, we drew the 3D structure of claudin-1 (Figure 3). We also assessed the possible surface accessibility of claudin-1 for these post translational modifications (Table 2). We found that Ser 56 and Thr 42, 153 and 167 were predicted as "Buried" i.e. not accessible for these types of modifications [40], while Ser 192, 205 and 206, and Thr 191 and 195 as "exposed" surfaces. This information depicts that Ser and Thr residues present at C-terminal have high access to these types of modifications.

We, therefore propose that phosphorylation and O-*β*-GlcNAc modifications on cytoplasmic C-terminal Ser and Thr residues of claudin-1 control the TJs functionality. Blocking the phosphorylation by O-*β*-GlcNAc modification on Ser 192, 205, 206, and Thr 191 and 195 can disrupt the binding of claudin-1 with other cytoplasmic proteins and reduce its cooperation during HCV viral entry. Furthermore, these potential phosphorylation sites of claudin-1 do present themselves as attractive candidates for novel chemotherapeutic agents, resulting in the halting HCV entry in the host cells.

## Abbreviations

HCV: Hepatitis C virus; CD81: cluster of differentiation 81; TJ: Tight junctions.

## Competing interests

The authors declare that they have no competing interests.

## Authors' contributions

WA and KS contributed equally to this study. WA, KS and SH designed the study. SA, SG, HK, KF, MTS and IS analyzed the data and wrote paper. All work was performed under supervision of SH. All authors read and approve the final manuscript.

## Authors' information

Bushra Ijaz (M Phil Molecular Biology), Waqar Ahmad (M Phil Chemistry) and Gull S (MSc Biochemistry) are Research Officer; Shabbiri K is lecturer while Fouzia K is BS (Hons) student at GC University, Lahore. Kausar H, Sawar MT and Shahid I are Phd scholars. Asad S is MPhil scholar, while Sajida Hassan (PhD Molecular Biology) is Principal Investigator at CEMB, University of the Punjab, Lahore
